# Detection of Crack Sealant in the Pretreatment Process of Hot In-Place Recycling of Asphalt Pavement via Deep Learning Method

**DOI:** 10.3390/s25113373

**Published:** 2025-05-27

**Authors:** Kai Zhao, Tianzhen Liu, Xu Xia, Yongli Zhao

**Affiliations:** 1 School of Transportation, Southeast University, Nanjing 210096, Chinaxiaxu1202@126.com (X.X.); 2 Jinan City Planning and Design Institute, Jinan 250101, China

**Keywords:** hot in-place recycling, detection, pavement, crack sealant, deep learning

## Abstract

Crack sealant is commonly used to fill pavement cracks and improve the Pavement Condition Index (PCI). However, during asphalt pavement hot in-place recycling (HIR), irregular shapes and random distribution of crack sealants can cause issues like agglomeration and ignition. To address these problems, it is necessary to mill large areas containing crack sealant or pre-mark locations for removal after heating. Currently, detecting and recording crack sealant locations, types, and distributions is conducted manually, which significantly reduces efficiency. While deep learning-based object detection has been widely applied to distress detection, crack sealants present unique challenges. They often appear as wide black patches that overlap with cracks and potholes, and complex background noise further complicates detection. Additionally, no dataset specifically for crack sealant detection currently exists. To overcome these challenges, this paper presents a specialized dataset created from 1983 pavement images. A deep learning detection algorithm named YOLO-CS (You Only Look Once Crack Sealant) is proposed. This algorithm integrates the RepViT (Representation Learning with Visual Tokens) network to reduce computational complexity while capturing the global context of images. Furthermore, the DRBNCSPELAN (Dilated Reparam Block with Cross-Stage Partial and Efficient Layer Aggregation Networks) module is introduced to ensure efficient information flow, and a lightweight shared convolution (LSC) detection head is developed. The results demonstrate that YOLO-CS outperforms other algorithms, achieving a precision of 88.4%, a recall of 84.2%, and an mAP (mean average precision) of 92.1%. Moreover, YOLO-CS significantly reduces parameters and memory consumption. Integrating Artificial Intelligence-based algorithms into HIR significantly enhances construction efficiency.

## 1. Introduction

**Figure 1:**
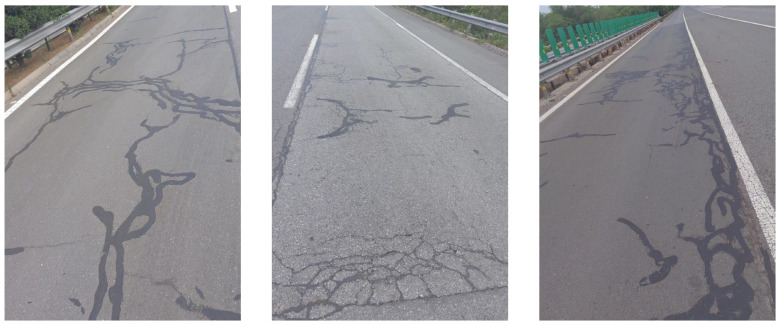
Crack sealant.

In the northwest region of China, asphalt pavement cracks are frequent and diverse [1]. Historically, due to economic constraints, crack filling has been a common treatment method as shown in Figure 1. Crack sealant, a material used for repairing cracks, is typically heated to a high temperature recommended by the manufacturer (usually around 193 °C) to become liquid, ensuring good adhesion and durability with the crack edges [2,3]. By filling the cracks, the pavement condition index (PCI) can be significantly improved in a short time. However, the lifespan of this treatment is generally short. Currently, the widespread use of hot in-place recycling (HIR) technology for recycling the surface layer of asphalt mixtures presents significant challenges when dealing with crack sealant [4,5,6]. During the heating process of old pavements, the variability in temperature due to uneven heating by the heating machine can be substantial. If the heating temperature is insufficient, the crack sealant does not revert to a liquid state but instead forms agglomerates [7]. For small areas of crack sealant, these agglomerates can be manually removed after heating, but this significantly slows down the construction process. However, if large areas of crack sealant are not pre-milled, repeated heating by the machine can cause the sealant (asphalt-based above 204 °C, silicone-based above 300 °C) to ignite, posing safety hazards. Therefore, accurately and effectively locating the position, type, and distribution of crack sealant during the pre-treatment process of origin pavements in HIR is crucial.

Traditional manual survey methods are still commonly used in HIR engineering to record the location and type of crack sealant through full-line inspections. Although accurate, these methods are influenced by subjective factors, time-consuming, and labor-intensive [8]. The results can vary significantly among technicians, and the process may cause traffic congestion and pose risks to survey personnel. To overcome these challenges, modern equipment is essential for capturing high-resolution pavement images. Line scan cameras, known for their high resolution, stable imaging quality, and low cost, are widely used in road detection. For example, Xiong et al. used a pavement inspection vehicle equipped with two line-scanning cameras, each capturing a line image of 2048 × 1 pixels [9]. To address traffic congestion and the difficulty of observing long longitudinal cracks, Wang et al. [10] used UAV (Unmanned Aerial Vehicle) oblique photography technology to collect high-resolution pavement images and perform 3D reconstruction. Similarly, Zhang and Zhu used the UACV (Unmanned Aerial Camera Vehicle) method to construct a pavement distress database for non-destructive testing [11,12]. However, after obtaining pavement images using these methods, manually classifying and locating the crack sealant on a computer remains costly and inefficient.

In the field of computer vision, object detection tasks identify objects of interest in images, determining their categories and locations [13,14]. Applying this technology to detect crack sealant on asphalt pavements is crucial. Traditional object detection algorithms, such as thresholding, edge detection, region growing, and clustering, rely on object features like color, shape, and texture [13]. However, these methods’ accuracy can be compromised when target shapes are complex, occluded, or have strong background noise. Convolutional Neural Networks (CNNs) have significantly advanced deep learning applications in object detection by learning the mapping between inputs and outputs without precise mathematical equations [15]. Deep learning-based object detection algorithms are categorized into two-stage and one-stage methods [16,17]. Two-stage methods involve feature extraction, region proposal (RP) generation, and classification/location regression. Representative algorithms include R-CNN, SPP-Net, Fast R-CNN, Faster R-CNN, and R-FCN. Matarneh et al. [18] introduced a method for asphalt pavement crack classification using the DenseNet201 model and GWO optimizer, achieving 98.73% accuracy and good robustness. Liang et al. [19] proposed a detection method based on Faster R-CNN to automatically identify and locate pavement issues such as cracks, potholes, and asphalt spills. Although these region-based two-stage models are highly accurate, their detection speed is generally slow, making them unsuitable for fast and lightweight pavement detection [20]. One-stage methods skip the RP step and directly extract features in the network to predict the classification and location of objects. Notable algorithms include OverFeat, YOLO, SSD, and RetinaNet. YOLOv1, proposed by Redmon in 2016 [21], significantly improved detection speed by transforming the object detection task into a regression problem. YOLO has been updated to version 10 and is widely used in pavement detection [22,23]. Several studies have compared different YOLO series models to identify the most suitable ones for pavement defect detection. For instance, Yao et al. improved YOLOv5 by incorporating the Space and Channel Squeeze-and-Excitation (SCSE) module and the Convolutional Block Attention Module (CBAM), considering different addition positions and methods [24]. Comparative tests achieved a detection speed of 87% mAP@0.5:0.95 and 13.15 ms/pic. Zhu et al. trained UAV datasets using Faster R-CNN, YOLOv3, and YOLOv4 models, finding YOLOv3 performed best with a mAP of 56.6% [12]. Liu et al. proposed YOLO-SST based on YOLOv5, introducing the Shuffle attention mechanism and an additional detection layer. Ablation experiments and comparative tests showed that YOLO-SST increased accuracy by 1.2% and mAP by 3.1% [25]. Researchers have conducted extensive studies on rutting, potholes, cracks, and ground-penetrating radar images, establishing high-quality datasets and improving detection accuracy and speed through deep learning algorithms.

In summary, detecting and identifying crack sealant involves several challenges: (1) Crack sealant is typically a wider black patch, while cracks are usually narrower [26,27]. This difference affects the accuracy and robustness of detection algorithms. (2) Crack sealant often overlaps with cracks and potholes, and background noise further complicates pavement detection. (3) No researchers have established a high-quality dataset specifically for crack sealant [8,28]. Therefore, existing pavement distress detection algorithms are not suitable for crack sealant detection, leading to high rates of missed and false detections, slow detection speeds, and large memory usage. These issues make it difficult to meet the requirements for lightweight detection and mobile deployment in engineering applications. This study aims to address these challenges and achieve high-precision crack sealant detection during the HIR pre-treatment process. The approach involves several steps: First, high-resolution full-scale pavement images are collected using a detection vehicle equipped with two line-scanning cameras. Images containing crack sealant are then selected and cut to create a dataset. Next, based on the YOLOv8s algorithm, lightweight improvements are made using RepViT, DRBNCSPELAN, and LSC detection heads. Finally, the proposed improvements are verified through ablation and comparative tests, with the results visualized and analyzed.

## 2. Methodology

### 2.1. YOLOv8s Network Model

The object detection benchmark model selected in this study is YOLOv8, developed by the Ultralytics team, and known for its cutting-edge and advanced features [22,29]. YOLOv8 introduces new functions and improvements that enhance performance and flexibility. Compared to other one-stage detection models and previous versions of the YOLO series, YOLOv8 demonstrates superior performance in detecting pavement distress [30,31,32,33]. YOLOv8 offers five network structure models: YOLOv8n, YOLOv8s, YOLOv8m, YOLOv8l, and YOLOv8x, as shown in Table 1. Each is tailored to different deployment scenarios, ranging from resource-constrained embedded devices to high-performance GPU servers. This paper aims to improve accuracy while maintaining high processing speed, making YOLOv8s the chosen benchmark model for further enhancement.

**Figure 2:**
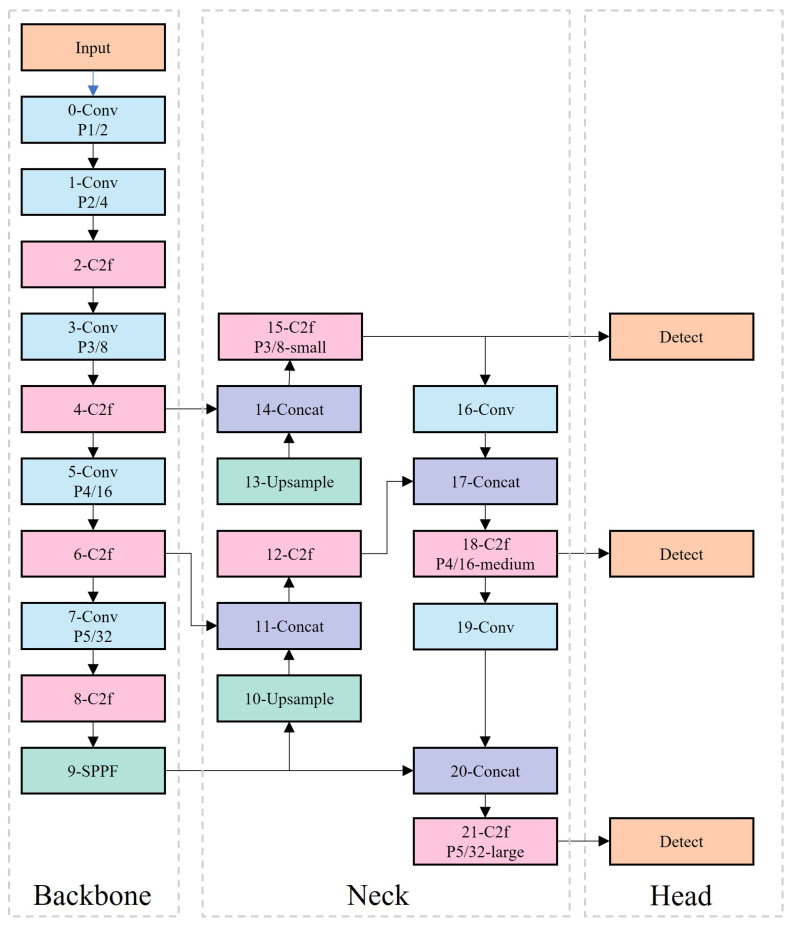
YOLOv8s network model. Note: Conv (Convolution); C2f (CSP-to-Fusion).

The network structure of YOLOv8s, shown in Figure 2, consists of four main parts: Input, Backbone, Neck, and Head.

The Backbone is the network component responsible for extracting image features, transforming the original input image into a multi-layer feature map for subsequent target detection tasks. YOLOv8 employs the C3 module and ELAN design principles to create a C2f structure, which ensures lightweight operation while capturing richer gradient flow information [34,35]. The Neck component handles multi-scale feature fusion of the feature maps and passes these features to the prediction layer. YOLOv8 uses PAN-FPN, mimicking the Backbone in the PANet for the Neck part [36,37]. This involves organizing the FPN (feature pyramid network) with both down-sampling and up-sampling processes [38]. There are two cross-layer fusion connections between the up-sampling and down-sampling branches. The Head performs the final regression prediction. YOLOv8 employs a decoupled-head structure for separate regression learning of categories and bounding boxes, adopting the Anchor-Free concept [39].

### 2.2. Overview of YOLO-CS

**Figure 3:**
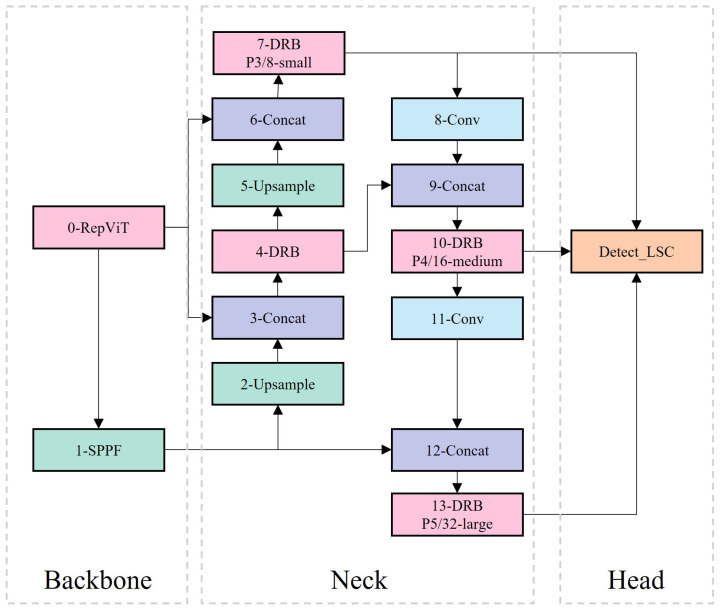
YOLO-CS network model.

This paper aims to achieve lightweight detection of sealant in the pretreatment process of HIR. To this end, a series of innovative improvements have been made to the YOLOv8s model as shown in Figure 3. Firstly, the RepViT backbone framework has been introduced, which enhances detection accuracy and reduces model complexity, thereby effectively decreasing computation time. RepViT enables the model to better capture feature information in the image, thus improving detection accuracy and achieving higher efficiency without additional computational burden.

Next, to further optimize model performance, the fusion of the Dilated Reparam Block (DRB) and Generalized ELAN has been employed to form the DRBNCSPELAN module. This module aims to enhance detection task performance by combining the benefits of DRB and Generalized ELAN. DRB expands the model’s receptive field through dilated convolution, helping to better capture long-range dependencies in the image, thereby improving detection accuracy. Generalized ELAN effectively handles fuzzy boundary conditions in specific scenarios, further enhancing the model’s robustness and generalization ability. By combining these two elements, the model better adapts to detection tasks in various scenarios, achieving superior performance.

Finally, to address the issue of the YOLOv8s detection head’s high computational demand, the LSC detection head has been developed. The LSC detection head uses a shared convolution design, which minimizes computational load and maintains accuracy by sharing the convolution kernel between multiple detection layers. This shared convolution layer extracts common feature information and shares it across multiple detection layers, reducing redundant calculations and lowering the model’s computational overhead. This design improves the model’s inference speed and efficiency while maintaining high detection accuracy, making the model more suitable for practical applications. Through these improvements and optimizations, YOLO-CS has significantly enhanced performance in the lightweight detection task of crack sealant, providing a more reliable and efficient solution for project applications.

### 2.3. RepViT Backbone Framework

**Figure 4:**
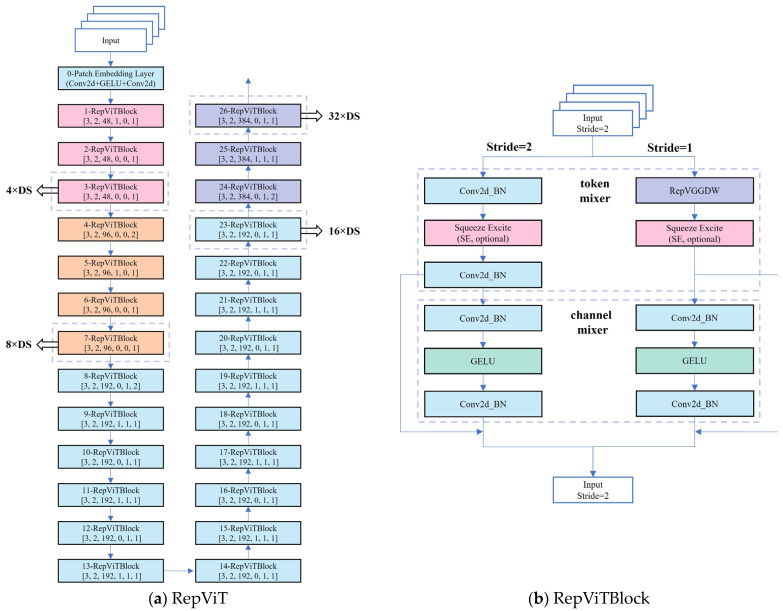
Overview of RepViT architecture.

While YOLOv8 demonstrates strong performance in accuracy and speed, its computational complexity and parameter quantity may result in slow reasoning speed and high power consumption on resource-constrained devices such as mobile or embedded devices. Additionally, YOLOv8s relies on the Darknet-53 backbone framework, which includes 52 layers of convolution plus an output layer, for detecting asphalt pavement sealant. However, this high structural complexity can limit its flexibility in deployment. Furthermore, YOLOv8’s universal design may not be as effective as specially designed lightweight models when handling specific tasks. Particularly with high-resolution input images, YOLOv8 might struggle to capture small and intricate sealant features. To address these limitations, this paper introduces the RepViT network architecture in Figure 4. Drawing inspiration from the Transformer and ViT (Vision Transformer) concepts, RepViT leverages self-attention mechanisms and global feature modeling to capture global context information in road images, thereby enhancing detection accuracy and robustness [40].

RepViT efficiently extracts both local and global features through a combination of token mixer and channel mixer. The Token Mixer employs deep convolution and point-by-point convolution for local feature extraction, enhancing the representation of local features. Meanwhile, the Channel Mixer facilitates the mixing and enhancement of different channels through point-by-point convolution and residual connection, ensuring information flow across scales and channels to capture more details and context information. Moreover, the modular design of RepViTBlock enables the model to dynamically adjust the number of layers and channels based on specific task requirements and resource constraints. This approach not only enhances the model’s adaptability but also optimizes its performance for diverse needs. Specifically, when the stride is set to 2, the model incorporates a depthwise separable convolution, Squeeze-and-Excite module, and pointwise convolution. Conversely, when the stride is 1, the RepVGGDW module is utilized for deep convolution operations along with the Squeeze-and-Excite module. This flexible design allows for the adjustment of model complexity and computational requirements while maintaining high performance.

### 2.4. DRBNCSPELAN Feature Fusion Module

**Figure 5:**
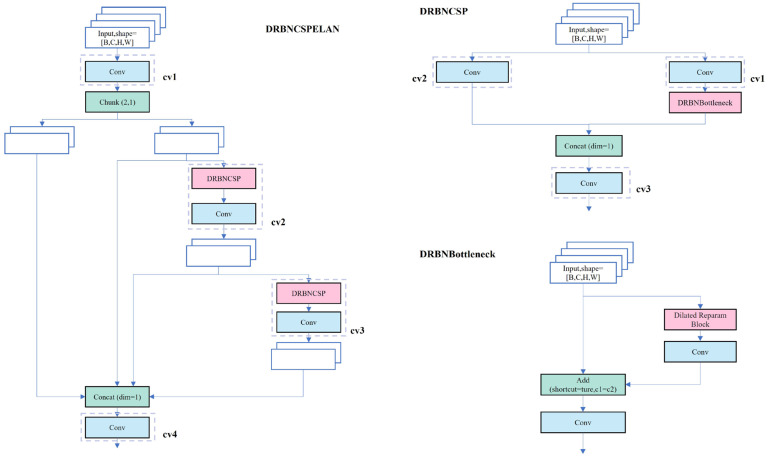
DRBNCSPELAN.

While the C2f module in YOLOv8s effectively leverages both detailed and semantic information for enhanced accuracy and robustness in pavement distress detection, its feature fusion operation escalates computational complexity and parameter count. Consequently, this elevates the time cost associated with model training and reasoning. To address this challenge, this paper introduces the DRBNCSPELAN module, replacing the C2f module, utilizing the Dilated Reparam Block and Generalized ELAN, as depicted in Figure 5.

**Figure 6:**
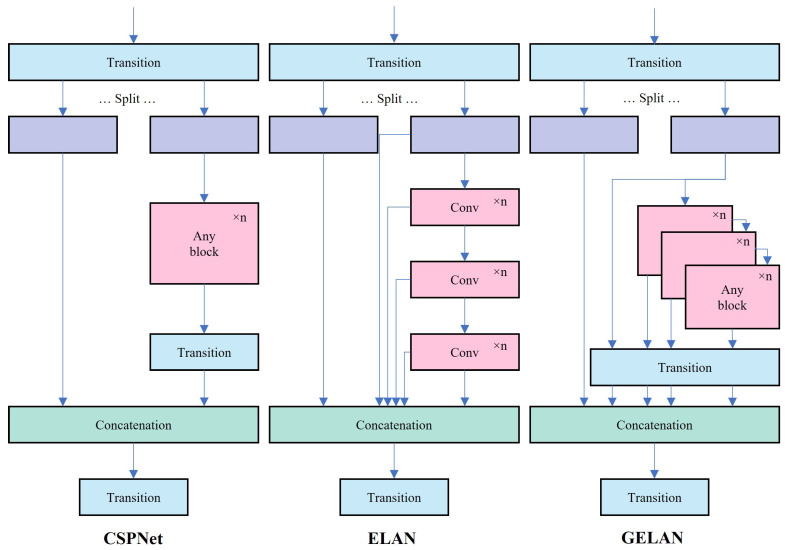
GELAN.

CSPNet is a network built upon stage-level gradient path-based architecture [41]. It divides the input into two segments via the conversion layer, processes them through any computational block, and subsequently reunites the branches through concatenation before passing them through the conversion layer again. Meanwhile, ELAN, a gradient path-oriented network, enhances the network’s gradient length by employing a stack structure within its blocks [42]. Through stacked convolution layers, each layer’s output is combined with the input of the subsequent layer for convolution processing. GELAN, inspired by CSPNet’s segmentation and reassembly concept and ELAN’s hierarchical convolution processing, integrates these elements into its design, allowing flexible utilization of computational blocks as shown in Figure 6 [23]. By facilitating efficient information flow and optimizing parameter utilization, GELAN reduces computing resource requirements while potentially enhancing detection accuracy and model generalization.

**Figure 7:**
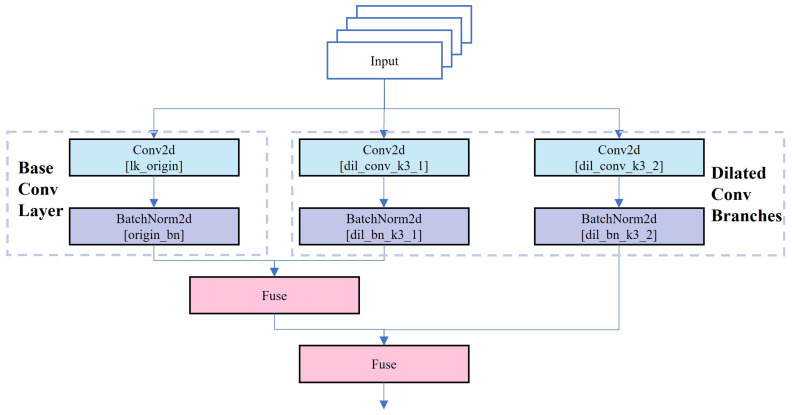
DRB.

The DRB (Dilated Reparam Block) aims to enhance model performance by combining large-kernel convolutions with dilated small-kernel convolutions as shown in Figure 7 [43].

It captures fine-scale features via parallel small-core convolutional layers and sparse features via dilated convolutional layers, thereby enriching feature extraction efficiency. During training, these parallel branch convolution layers are each batch normalized (BN), and their outputs are aggregated. In the inference stage, structural reparameterization combines these convolutional layers and batch normalization layers into an equivalent large kernel convolutional layer, reducing computational overhead. A notable innovation of this module is converting dilated convolutional layers into non-expansive sparse large kernel convolutional layers. Specifically, by introducing zero entries into the convolution kernel, expanded convolution layers can be transformed into sparse non-expanded large kernel convolution layers. This approach preserves the effectiveness of original dilated convolutions while simplifying calculations during inference, achieved through transpose convolution. The integration of BN layers and dilated convolution layers enables the entire DRB to be converted into a single non-expanded large kernel convolution layer during inference, significantly boosting inference speed while maintaining efficient feature extraction.

### 2.5. Lightweight Shared Convolutional Detection Head

**Figure 8:**
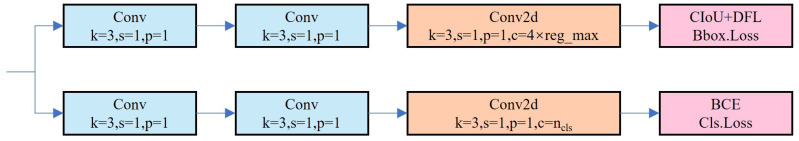
YOLOv8s decoupled head.

The Head part of YOLOv8s comprises two CBS convolution structures bifurcated, followed by a Conv2d operation as shown in Figure 8. Subsequently, classification loss and Bbox loss are computed separately. YOLOv8s adopts the Decoupled-Head structure to segregate classification and detection heads. Moreover, inspired by the Distributional Focal Loss (DFL) concept, the regression head’s channel count becomes 4 × reg_max (defaults: 16). Despite enhancing detection accuracy, the independent convolution operation employed for each detection layer’s feature map processing leads to redundant computational overhead. This approach underutilizes shared information within the feature map, thereby increasing the model’s computational cost. Additionally, during inference, the need to concatenate multiple convolution outputs and perform intricate post-processing escalates computational complexity and memory usage.

**Figure 9:**
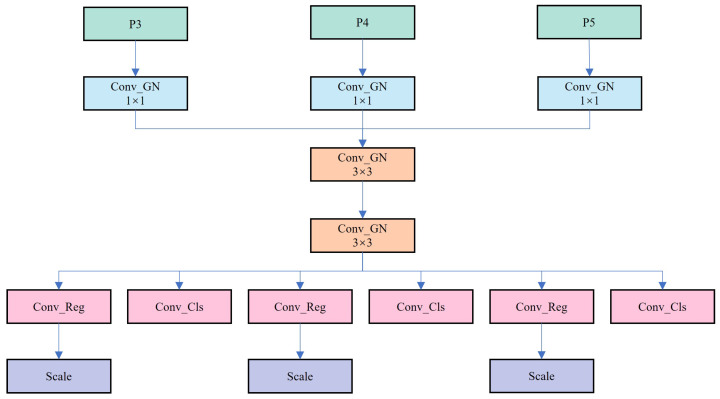
LSC detection head.

To enhance detection efficiency, this paper proposes a Lightweight Shared Convolutional (LSC) Detection Head, outlined in Figure 9.

This structure enhances model performance and efficiency by amalgamating shared and independent convolutional layers, along with utilizing the distributed focus loss (DFL) module. The crux of its design lies in integrating shared convolutional layers, composed of two 3 × 3 convolutional layers for extracting general feature information, and independent convolutional layers, housing multiple 1 × 1 convolutional layers for regression and classification output. GroupNorm, proven effective in enhancing detection head performance, replaces the normalization layer BN in Conv to counteract feature extraction capability weakening in lightweight scenarios [44]. This design facilitates shared common feature extraction among multiple detection layers, thereby curtailing redundant calculations, lowering computational overhead, and preserving high detection accuracy. Introducing a distributed focus loss (DFL) module further enhances bounding box regression accuracy, facilitating more precise object localization in practical applications.

Beyond structural optimization, the LSC boasts significant advantages in forward propagation. The presence of shared convolution layers streamlines the model’s forward propagation path, reducing concatenation and post-processing steps, and thereby boosting reasoning speed and efficiency. This streamlined forward propagation path not only accelerates model inference but also diminishes computing resource requirements, rendering the model more suitable for real-time scenarios. To address inconsistent target scales across detectors, the Scale layer is employed alongside shared convolution. In summary, LSC not only boosts inference speed and efficiency while maintaining detection accuracy but also excels in resource-constrained environments, promising broad application potential.

## 3. Dataset Construction and Experiment Setup

### 3.1. Dataset Construction

**Figure 10:**
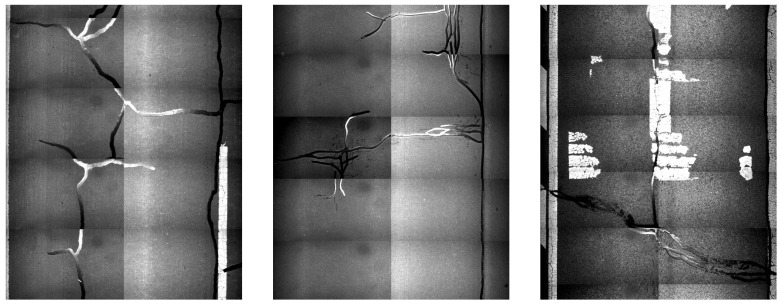
Pavement full-scale image.

To verify the effectiveness of the proposed YOLO-CS in accurately locating and classifying pavement crack sealant, a high-quality dataset is essential. Current public datasets primarily focus on various pavement cracks, pits, and repairs, often lacking sufficient clarity. To address this, a pavement detection vehicle LUPRES-T equipped with two line-scanning cameras was used to capture a 2512 × 3140 pixel image as shown in Figure 10. These images were taken on a high-speed maintenance section in Northwest China, with fully enclosed construction ensuring the detection vehicle traveled at a constant speed of 40 km/h during shooting.

Since the input image size for YOLOv8s is 640 × 640 pixels, directly inputting the original image would result in excessive downscaling, leading to loss of detail and impairing accurate classification and positioning. Therefore, the original image was proportionally cropped to 628 × 628 pixels. A total of 1983 pavement images containing transverse, longitudinal, and block sealants were selected, as detailed in Table 2.

Using Make Sense software (https://www.makesense.ai/), various sealants were marked with rectangular bounding boxes in the asphalt pavement images. The marking information, including type, center point coordinates, width, and height, was saved directly in the text format compatible with the YOLO model. After labeling and expert review, the dataset comprised 1983 road images and 2447 labeled crack sealants. The dataset was randomly divided into training, validation, and test sets in a 7:1:2 ratio, as shown in Table 3. This methodology ensures a robust and precise dataset for evaluating the performance of YOLO-CS.

### 3.2. Experiment Settings

The training, testing, and validation of the YOLO series are conducted on a computer workstation equipped with a 16 GB NVIDIA GeForce RTX 3060 GPU. The main training parameters of YOLO are as follows: Cache determines whether to use cache when loading data to speed up the reading process. In this paper, the cache is set to False. Epochs refer to the number of training rounds. More epochs allow the model to learn the data more thoroughly but also increase training time and risk overfitting. After extensive experimentation, this paper sets the epochs to 500. Batch size refers to the number of images in each batch. Larger batch size can better represent the data set distribution and improve model learning but requires more graphics memory. This paper sets the batch size to 16. Mosaic data enhancement enriches the background of images and improves the batch size through image splicing. In this paper, mosaic enhancement is disabled in the last 10 rounds of training (close mosaic is set to 10). Workers refer to the number of working threads used when loading data. This paper sets the number of workers to 8. Device refers to the hardware used for training. Since GPU is used to accelerate training, the device is set to 0. Optimizer refers to the algorithm used to adjust model parameters in deep learning to minimize the loss function. YOLOv8s offers several optimizers, including SGD, Adam, and RMSProp. This paper consistently uses the SGD optimizer. All remaining training parameters are left at their default settings.

### 3.3. Evaluation Metrics

FPS (Frames Per Second) indicates the number of images processed per second, used to evaluate the model’s processing speed on a given hardware setup. FLOPs (Floating Point Operations) measure the number of operations required to process an image, providing a hardware and software-independent metric.

Precision measures the proportion of correct positive predictions among all positive predictions, assessing the accuracy of the model in positive prediction scenarios. Recall measures the proportion of correct positive predictions among all actual positives, indicating the model’s ability to identify positive examples.

(1) $$Precision=\frac{TP}{TP+FP}$$

(2) $$Recall=\frac{TP}{TP+FN}$$

where: True Positive (TP) refers to correctly predicting the positive class. False Positive (FP) refers to incorrectly predicting the negative class as positive. False Negative (FN) refers to incorrectly predicting the positive class as negative.

Average Precision (AP) calculates the average accuracy for different categories:

(3) $$AP=\sum_{k=0}^{k=n-1}\left[Recalls(k)-Recalls(k+1)]\times Precisions(k\right)$$

where: Recalls(n)=0, Precisions(n)=1, n=Number of thresholds.

Mean Average Precision (mAP) is the average of APs across all categories. mAP50 represents the average mAP with an Intersection over Union (IOU) threshold greater than 0.5, while mAP50-95 represents the average mAP at various IOU thresholds, ranging from 0.5 to 0.95 in steps of 0.05.

(4) $$mAP=\frac{1}{n}\sum_{k=1}^{k=n}AP_{k}$$

where: $AP_{k}=the\;AP\;of\;class\;k$, n=Number of classes.

## 4. Result and Discussion

### 4.1. Algorithm Ablation Experiment

To investigate the impact of RepViT, DRBNCSPELAN, and LSC detection heads on the performance of YOLOv8s, ablation experiments were conducted. The results are presented in Table 4. Using YOLOv8s as the benchmark model, a “√” tick symbol indicates the inclusion of the corresponding module. Introducing the RepViT backbone network structure, which captures global context information in pavement images, resulted in Precision increasing by 4%, Recall by 3.9%, mAP50 by 3.8%, and mAP50-95 by 7%. The DRBNCSPELAN module, based on the innovative fusion of the Dilated Reparam Block and Generalized ELAN, improves information flow and the model’s generalization ability compared to the C2f feature fusion module. Precision increased by 6%, Recall by 1.6%, mAP50 by 2.8%, and mAP50-95 by 8.9%. Additionally, the number of parameters decreased from 11,126,745 to 7,666,569, significantly reducing the model’s memory usage to just 15.2 MB. The LSC detection head, developed to enhance detection performance with fewer parameters and computations, resulted in a 4.6% increase in Precision, a 3.2% increase in Recall, and increases of 3.9% in mAP50 and 7.4% in mAP50-95. Furthermore, the parameters and memory were reduced to 84.7% of the original, and FPS increased to 156.4 f·s^−1^.

Combining these models further enhanced performance. The integration of the RepViT framework with DRBNCSPELAN and LSC detection heads increased mAP50 by 3.5% and 1.8%, respectively, and mAP50-95 by 7.6% and 5.2%, respectively. The combination of DRBNCSPELAN and the LSC detection head achieved the minimum number of parameters (5,970,054) and memory usage (11.9 MB), with FLOPs at only 16.9. Additionally, mAP50 and mAP50-95 increased by 3.1% and 7.6%, respectively. The optimal improvement was achieved by combining all three strategies. This approach increased Precision by 5.4%, Recall by 1.7%, mAP50 by 4%, and mAP50-95 by 9.1%, while reducing parameters and memory by 30.2% and 27.9%, respectively. These improvements facilitate model lightweighting and significantly enhance the detection performance for asphalt pavement crack sealants. These ablation experiment results confirm the effectiveness of the proposed improvement strategies.

### 4.2. Model Comparative Analysis

To further evaluate the performance of the model, this study compares the YOLO-CS model with commonly used YOLO series models (YOLOv3-tiny, YOLOv5s, YOLOv6s, YOLOv8s) on the same asphalt pavement crack sealant dataset. The test results (Table 5) are as follows:

The YOLO-CS model outperforms other YOLO series models in detecting asphalt pavement crack sealant. YOLO-CS achieves 88.4% Precision, 84.2% Recall, 92.1% mAP50, and 71.2% mAP50-95. Additionally, the model’s FLOPs and size are 23.2 and 15.5 MB, respectively. Compared to YOLOv5s, YOLOv6s, and YOLOv8s, YOLO-CS’s mAP50 increased by 1.8%, 1.8%, and 4%, respectively, while mAP50-95 increased by 6.8%, 2.7%, and 9.1%, respectively. Furthermore, YOLO-CS has the smallest number of parameters and memory footprint, with only 7,764,542 parameters and a size of 15.5 MB. This makes it highly suitable for lightweight deployment under resource-constrained conditions. In summary, by utilizing the RepViT backbone network architecture and the DRBNCSPELAN feature fusion module, combined with the self-developed LSC lightweight detection head, the study significantly enhances high-performance detection of crack sealant during HIR while greatly reducing the number of parameters and memory usage.

### 4.3. Visualization Analysis

**Figure 11:**
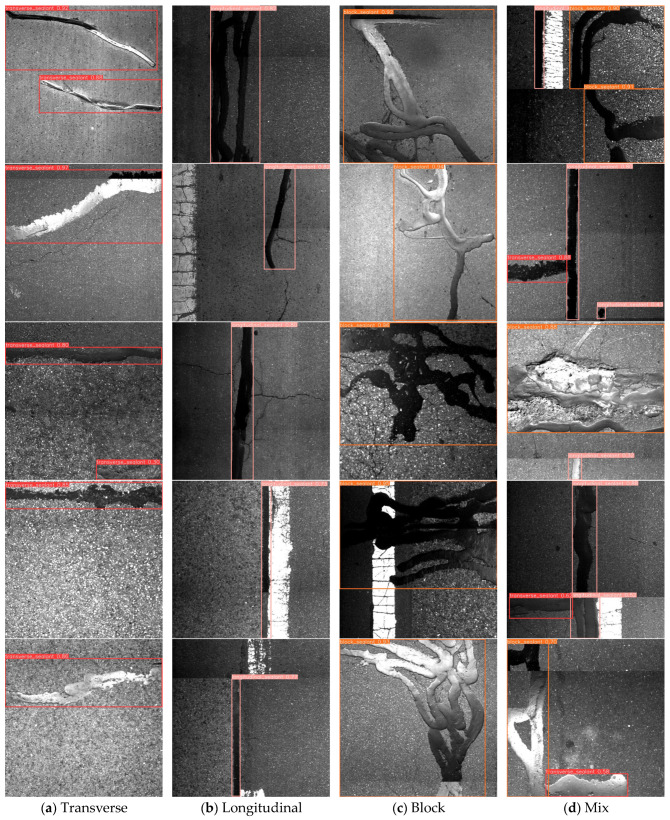
YOLO-CS visualization results.

To assess the practicality of the YOLO-CS model in asphalt pavement HIR pretreatment, a visual analysis of crack sealant detection was conducted, as depicted in Figure 11. In Figure 11a, most of the transverse crack sealant detections exhibit confidence levels above 0.8. Notably, the micro-surface pavement enhances detection accuracy due to better background distinction. This type of crack sealant is typically employed for low-temperature transverse crack repairs or reflective cracks, with a wide distribution interval, making excavation and collection straightforward. Figure 11b illustrates the detection of longitudinal sealant, with confidence ranging from 0.7 to 0.9, effectively distinguished from cracks. This sealant addresses fatigue longitudinal or minor network cracks and is suitable for expansive construction cracks, requiring similar treatment measures to transverse sealant. In Figure 11c, block sealant is predominantly detected with confidence exceeding 0.9, largely unaffected by the pavement background. This type often necessitates milling during pretreatment due to structural pavement damage, where HIR alone cannot restore mixture performance. The dense distribution of such sealant poses manual handling challenges and risks fire hazards during heating. Figure 11d exhibits a mixed distribution of various sealant types. YOLO-CS accurately and promptly identifies and classifies sealants across diverse pavement backgrounds, significantly reducing human resource requirements. Its lightweight design facilitates deployment on mobile terminals, fostering engineering applications.

**Figure 12:**
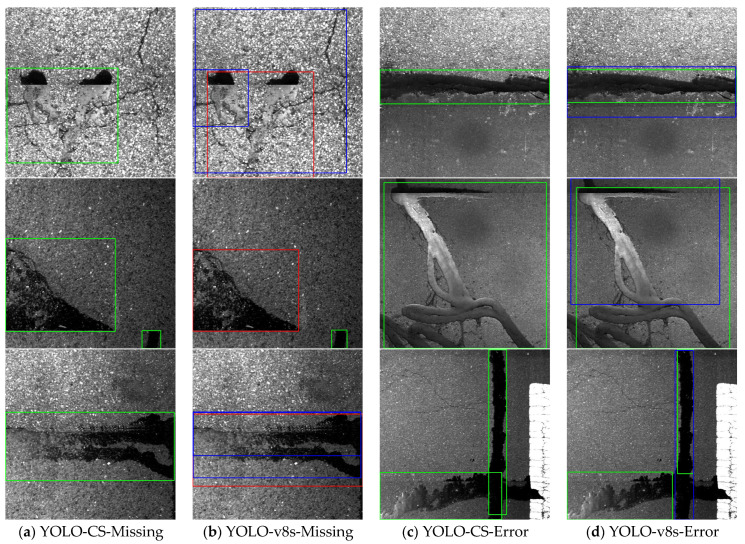
Visualization comparison between YOLO-CS and YOLOv8s.

To further compare the advantages of YOLO-CS over the YOLOv8s, the test set is analyzed. In Figure 12, correct detections are indicated by green boxes, missed detections by red boxes, and false detections by blue boxes. Analysis of Figure 12a,b reveals that when the surface lacks micro-surfaces, leading to low background discrimination, YOLOv8s struggles to differentiate cracks from block grouting glue, resulting in missed detections. YOLOv8s also misses bulk crack sealant in the second row and generates multiple prediction frames for transverse crack sealant, leading to missed detection. Conversely, YOLO-CS accurately locates the position and type of crack sealant. In Figure 12c,d, false detections in YOLOv8s are attributed to multiple prediction boxes for the same crack sealant, resulting in erroneous detection. YOLO-CS demonstrates superior learning and detection capabilities under varied conditions, accurately locating crack sealant positions with high confidence.

The statistical results of the test set are presented in Table 6, revealing a 14% decrease in missed detection rate and an 18% decrease in false detection rate with YOLO-CS. This underscores how the proposed YOLO-CS algorithm enhances detection accuracy and efficiency while achieving lightweight detection of crack sealant, effectively addressing challenges like strong background noise and multi-type target occlusion.

## 5. Conclusions

To achieve lightweight and precise positioning and classification of crack sealant during the pretreatment of hot in-place recycling for asphalt pavement, this paper introduces YOLO-CS, based on YOLOv8s. Firstly, it replaces Darknet-53 with the RepViT lightweight backbone network structure, which effectively reduces the convolutional neural network’s parameters and better processes long-range dependencies in images through a self-attention mechanism and global feature modeling. Secondly, it fuses the Dilated Reparam Block and GELAN to create the DRBNCSPELAN module, replacing the C2f feature fusion module to optimize parameter utilization and facilitate efficient information flow. Finally, a Lightweight Shared Convolutional (LSC) detection head is developed to enhance reasoning speed and efficiency while maintaining detection accuracy through shared convolution. Compared to the YOLOv8s benchmark model, YOLO-CS achieves a 5.4% increase in Precision, 1.7% increase in Recall, and improvements of 4% and 9.1% in mAP50 and mAP50-95, respectively, with a reduction in parameters and memory consumption by 30.2% and 27.9%, respectively. Its FPS is only slightly decreased. This model exhibits clear advantages over existing YOLO series object detection algorithms while maintaining superior detection performance and achieving lightweight deployment.

Furthermore, this paper establishes a dataset of asphalt pavement crack sealant, comprising 1983 pavement images containing transverse, longitudinal, and block crack sealant, with micro-surface and non-micro-surface sections in the pavement background. The images are clear at 628 × 628 pixels. The YOLO-CS model trained on this dataset demonstrates broad applicability. In summary, the YOLO-CS model developed in this study addresses the reliance on manual recording for crack sealant detection in the current pretreatment process, significantly reducing costs and improving detection efficiency. Implementing appropriate treatment measures based on the detection of different types of crack sealant can enhance the performance of recycled pavement.

## 6. Future Work

In future research, it is essential to evaluate the YOLO-CS model’s detection efficiency under various external factors, such as the presence of water and snow on the pavement surface, and to assess the impact of weather conditions during image capture. Given that crack sealant may deteriorate 1–3 years after application due to climate and load conditions, it is crucial to study whether the sealant’s condition affects detection accuracy. Specifically, it is important to determine if the model can accurately detect severely deteriorated crack sealant (e.g., settlement versus adhesive and cohesive failures). Further expansion of the crack sealant dataset is necessary to enhance the model’s robustness. Additionally, the development of software or mobile apps will promote the practical application of this method in engineering projects, thereby improving on-site construction efficiency.

## Figures and Tables

**Table 1:** YOLOv8 series model.

Model	Depth	Width	Max Channel	Size (Pixels)
YOLOv8n (nano)	0.33	0.25	1024	640
YOLOv8s (small)	0.33	0.50	1024	640
YOLOv8m (medium)	0.67	0.75	768	640
YOLOv8l (large)	1.00	1.00	512	640
YOLOv8x (extra large)	1.00	1.25	512	640

**Table 2:** Types of crack sealant for asphalt pavement.

Type	Corresponding Figure			
Transversecrack sealant	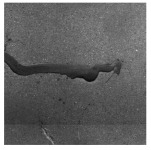	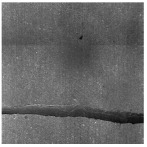	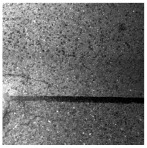	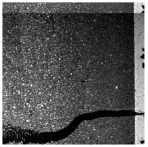
Longitudinalcrack sealant	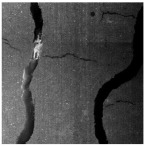	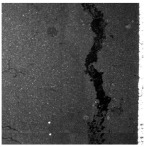	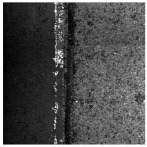	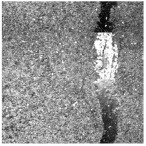
Blockcrack sealant	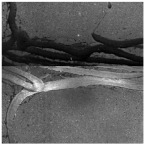	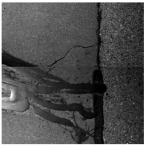	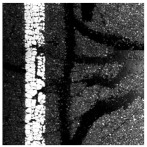	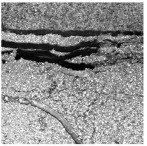

**Table 3:** Crack sealant dataset.

Dataset	Transverse Crack Sealant		Longitudinal Crack Sealant		Block Crack Sealant	
	Images	Objects	Images	Objects	Images	Objects
Training	554	581	536	582	528	552
Validation	85	87	71	76	72	72
Test	176	183	155	172	138	142
Sum	815	851	762	830	738	766

**Table 4:** Ablation study.

Baseline	Rep	DRB	LSC	P/%				R/%				mAP50/%					mAP50-95/%			
				TC	LC		BC	TC	LC		BC	TC	LC		BC		TC	LC		BC
YOLO v8s	-	-	-	85.8	84.9		78.4	92.2	78.6		76.8	95.4	84.6		84.2		64.2	56.5		65.6
	√	-	-	90.2	85.4		85.4	90.1	85.5		83.8	93.8	91.1		90.8		69.4	64.9		73.1
	-	√	-	92.3	87.3		87.4	91.3	83.7		77.5	94.5	90.4		87.9		71.5	65.6		76
	-	-	√	91	86.1		85.7	93.5	86.2		77.5	96.6	88.2		91.1		71.2	61.7		75.6
	√	√	-	92.9	84.3		90.9	89.1	82		77.2	94.6	89.2		91		69.5	63.2		76.4
	√	-	√	92.2	82.3		78.4	90.5	83.7		87.3	94.5	85.6		89.5		68.8	60.6		72.6
	-	√	√	93.6	85.5		84.3	86.9	81.4		83.8	94.5	88.7		90.5		70.5	63.1		75.5
	√	√	√	88.5	87.2		89.7	91.3	86		75.4	95.3	90.7		90.2		71.2	65.7		76.6
**Baseline**	**Rep**	**DRB**	**LSC**	**Precision**		**Recall**		**mAP50**		**mAP** **50-95**		**Param**		**Flops**		**Size**			**FPS**	
YOLO v8s	-	-	-	83		82.5		88.1		62.1		11,126,745		28.4		21.5 M			137.4	
	√	-	-	87		86.4		91.9		69.1		11,181,281		29.6		22.0 M			106.9	
	-	√	-	89		84.1		90.9		71		7,666,569		19.6		15.2 M			124.9	
	-	-	√	87.6		85.7		92		69.5		9,430,230		25.8		18.2 M			156.4	
	√	√	-	89.4		82.7		91.6		69.7		9,461,057		25.9		18.8 M			101.8	
	√	-	√	84.3		87.2		89.9		67.3		9,484,766		26.9		18.7 M			109.9	
	-	√	√	87.8		84		91.2		69.7		5,970,054		16.9		11.9 M			128.3	
	√	√	√	88.4		84.2		92.1		71.2		7,764,542		23.2		15.5 M			102.8	

Where: TC = Transverse crack sealant; LC = Longitudinal crack sealant; BC = Block crack sealant.

**Table 5:** Comparative experiments.

Model	Precision %	Recall %	mAP50 %	mAP50-95 %	Parameters	Flops	Size	FPS
YOLOv3tiny	59.5	71.8	67.8	29.0	12,129,206	18.9	23.2 M	232.9
YOLOv5s	86.9	82.7	90.3	64.4	9,112,697	23.8	17.7 M	157.4
YOLOv6s	86.2	84.7	90.3	68.5	16,298,009	44.0	31.4 M	131.4
YOLOv8s	83	82.5	88.1	62.1	11,126,745	28.4	21.5 M	137.4
YOLO-CS	88.4	84.2	92.1	71.2	7,764,542	23.2	15.5 M	102.8

**Table 6:** Test result.

	Right	Missing	Error (Misclassification)
YOLOv8s	445	42	172
YOLO-CS	449	36	141

## Data Availability

Dataset available on request from the authors.
